# Good long-term outcome of synovectomy in advanced stages of the rheumatoid elbow: 64 elbows followed for 10–23 years

**DOI:** 10.3109/17453674.2012.702391

**Published:** 2012-08-25

**Authors:** Katsushi Ishii, Yutaka Inaba, Yuichi Mochida, Tomoyuki Saito

**Affiliations:** ^1^Department of Orthopaedic Surgery, Yokohama City University; ^2^Center for Rheumatic Diseases, Yokohama City University Medical Center, Yokohama, Japan

## Abstract

**Background and purpose:**

Synovectomy is an effective procedure for management of the rheumatoid elbow at radiographically early stages (Larsen grades 1 and 2). However, its efficacy for advanced stages (Larsen grades 3–5) is controversial. We investigated the outcome of synovectomy for advanced stages of the rheumatoid elbow.

**Methods:**

Between May 1985 and September 1994, synovectomy was performed for 67 rheumatoid elbows in 59 patients (mean age 52 (26–72) years, 54 women). 3 elbows (3 patients) were lost to follow-up after mean 15 (10–23) years. Thus, 64 elbows were evaluated clinically and radiographically.

**Results:**

The mean Mayo elbow performance score (MEPS) improved from 42 (15–75) points preoperatively to 78 (45–100) points at the final follow-up examination. In cases of Larsen grade 5, the mean MEPS at final follow-up examination (69 points) was lower than those of Larsen grade 3 and 4 cases (80 and 79 points, respectively) (p < 0.01). Recurrence of synovitis was obvious in 20/67 elbows. 12 cases had a total elbow arthroplasty mean 13 years after the synovectomy. The 10-year, 15-year, and 20-year survival rates were 97%, 75%, and 70%, respectively.

**Interpretation:**

Our findings suggest that synovectomy for the rheumatoid elbow gives a good long-term outcome for radiographically judged destroyed joints of Larsen grades 3–4.

Recently, in addition to conventional disease-modifying anti-rheumatic drugs, biologic therapies have been widely introduced for the treatment of rheumatoid arthritis (RA). However, despite the existence of these treatments, there are many cases with high disease activity and continuous local synovitis in certain joints.

The elbow joint has high morbidity, followed by joints in the finger, the wrist, the toe, and the knee. The incidence of elbow joint destruction in RA patients is 20–50% (Souter 1990, Mansat 2001). Continuous synovitis may cause progressive joint destruction, which results in severe joint instability or contracture.

The local treatment of continuous synovitis includes physiotherapy and intraarticular injections of steroids. However, when the effects of these treatments are insufficient, surgical treatment is often recommended: synovectomy or total elbow arthroplasty (TEA). Synovectomy is often reported as a successful procedure with relatively few complications for the treatment of rheumatoid elbows at radiographically early stages (Larsen grades 1 and 2) (Stein et al. 1975, Brumfield and Resnick 1985, Saito et al. 1986, Ferlic et al. 1987, Tulp and Winia 1989, Koshino et al. 1991, Gendi et al. 1997, Fuerst et al. 2006). TEA is usually performed for joints at radiographically advanced stages (Larsen grades 3–5) (Figgie et al. 1989, Connor and Morrey 1998, Mansat and Morrey 2000). However, for cases with good ligament stability, we have even performed synovectomies for Larsen grades 3–5, which, however, is controversial (Nestor 1998).

In this study, we investigated the long-term outcome of synovectomy that was performed for the treatment of rheumatoid elbows at radiographically advanced stages.

## Patients and methods

From May 1985 to September 1994, we performed synovectomy for 67 rheumatoid elbows at radiographically advanced stages (59 patients, 54 women). 12 elbows were classified as grade 3, 45 as grade 4, and 7 as grade 5 according to Larsen’s classification. Mean age at surgery was 53 (26–72) years. 3 elbows in 3 patients were lost to follow-up; thus, data for 64 elbows of 56 patients were used in the study. The 64 elbows had an average follow-up period of 15 (10–23) years. Surgical indications of synovectomy were continuous pain that did not respond to nonoperative treatment or serious disability due to progressive loss of range of motion.

Functional outcome was assessed using the Mayo elbow performance score (MEPS) (Morrey 1991). Conventional anteroposterior and lateral radiographs were taken before surgery and at the final follow-up examination. The grades of Larsen’s classification and the elbow valgus angle before surgery and at the final follow-up examination were evaluated (Larsen et al. 1977).

### Surgical procedure and postoperative care

A laterally curved 10-cm skin incision was made in the posterolateral area of the elbow. Dissection was done between the brachioradialis and the triceps muscles in the upper arm and between the extensor carpi radialis and the anconeus in the forearm. The anterior capsule and the lateral ligament were exposed entirely. After resection of the radial head, the anterior joint capsule with the synovium was completely detached from the lateral to the medial condyle of the humerus. The synovium remaining around the capsular attachment to the humerus and around the radioulnar joint was resected with a small rongeur. After the posterolateral joint cavity was exposed, the synovectomy was performed by resecting the capsule adhering to the posterior side of the humerus and resecting the synovium in the posterolateral capsule and around the olecranon fossa. Because we used a lateral approach, most of the synovium in the medial capsule could not be resected. Osteophytes around the olecranon and the coronoid process, if present, were resected until full range of extension and flexion was achieved. A suction drain tube was inserted into the joint space for 24 h. No external fixation was used. Active exercises of the elbow were started on the day after surgery. To avoid flexion contracture, the elbow was gradually extended while a 0.5- to 1.0-kg weight was held in the hand. Home exercises were continued until enough range of motion was obtained.

### Statistics

We used Wilcoxon’s signed-rank test and chi-square test to compare the clinical results before surgery and at the final follow-up examination. Values of p less than 0.05 were considered significant. Survival analysis was performed by the Kaplan-Meier method, and the endpoint was defined as conversion to TEA.

## Results

### Clinical and radiographic outcome

**Figure 1.**
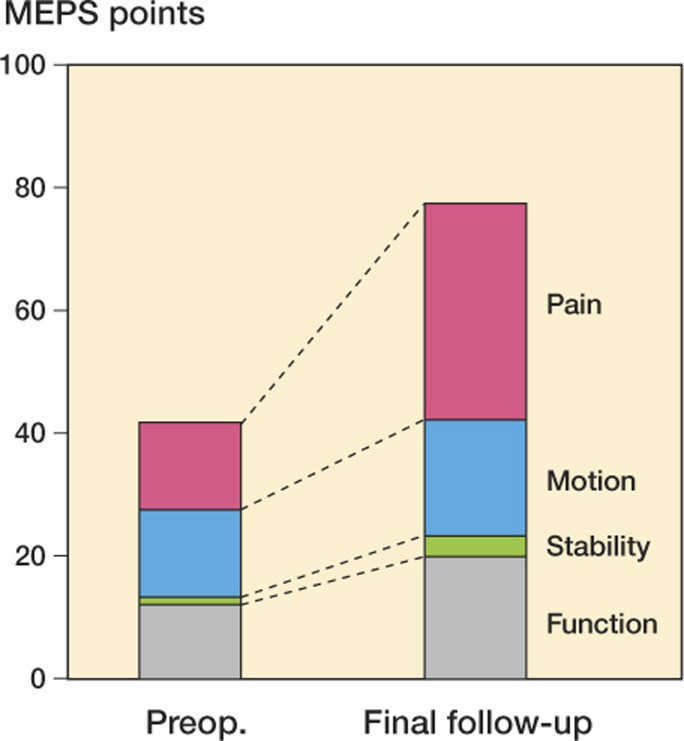
The total Mayo elbow performance score (MEPS) preoperatively and at the final follow-up.

**Figure 2.**
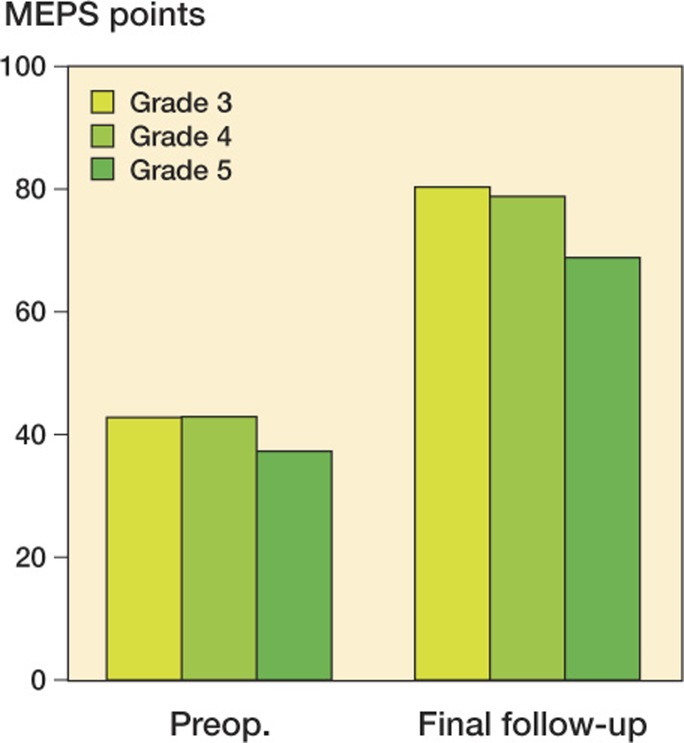
MEPS preoperatively and at the final follow-up examination. The mean scores improved statistically significantly for all grades.

According to the MEPS, 48 elbows showed excellent or good results, 12 elbows showed fair results, and 4 elbows had poor results at the final follow-up examination. The total MEPS improved from an average of 42 (15–75) points before surgery to 78 (45–100) points at the final follow-up examination (p < 0.01) (Figure 1). There were no cases that had lower MEPS at the final follow-up examination than preoperatively. For all elbows, MEPS was statistically significantly improved at the final follow-up examination (Figure 2).

**Figure 3.**
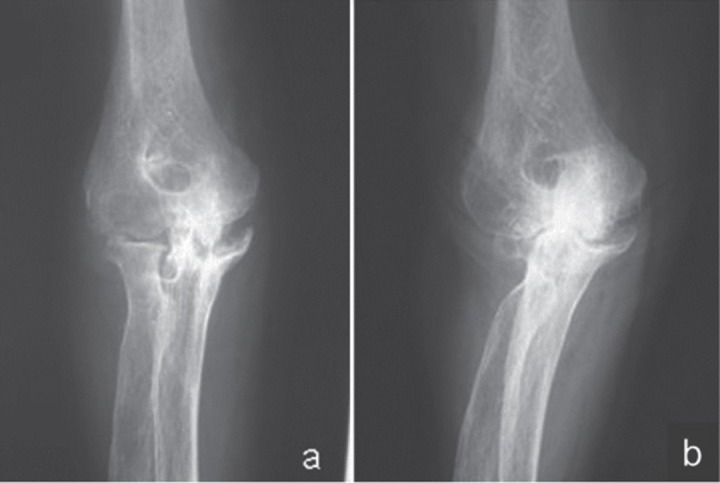
a. A right elbow 6 years after the onset of rheumatoid arthritis; Larsen grade 4. This patient complained of severe pain, swelling, and disability in activities of daily living (ADL), and motion was from 30° to 105°. The preoperative MEPS was 50 points. b. 11 years after surgery; Larsen grade 4. The patient had experienced relief of the pain with no swelling and no disability in ADL. The MEPS was 95 points.

The mean MEPS for pain improved from 14 (0–30) points before surgery to 35 (15–45) points after surgery. Complete pain relief or only mild pain (scores of 30–45) after the elbow synovectomy was observed in 62 cases (Figure 3). 2 elbows showed moderate pain at the final follow-up examination.

The mean arc of flexion-extension motion increased from 67° (0–125) preoperatively to 101° (65–140) postoperatively (p < 0.01) and from 102° (20–70) to 137° (80–180) in pronation and supination (p < 0.05). In 2 elbows, the arc of flexion-extension motion worsened postoperatively. In pronation and supination, 52 elbows had an improved arc of motion preoperatively, 10 elbows had a reduced arc of motion, and 2 elbows (3.1%) had the same arc of motion preoperatively. Regarding the arc of motion, the preoperative MEPS increased from 14(5–20) points to 19 (15–25) points at the final follow-up examination. The function score was 12 (0–20) points preoperatively and 20 (15–45) points at final follow-up. The changes in the total score at the final follow-up examination mainly depended on the relief of pain in 12 elbows that had been preoperatively graded as Larsen grade 3; of these, 4 elbows remained at grade 3, and 7 elbows and 1 elbow progressed to Larsen grade 4 and 5, respectively. In 45 elbows that had been graded as Larsen grade 4 preoperatively, 32 elbows remained at grade 4 and 13 elbows progressed to Larsen grade 5. Assessment of clinical mediolateral joint stability showed minimal instability (0–5°) in 59 elbows, moderate instability (6–10°) in 3 elbows, and marked instability (> 10°) in 2 elbows.

### Survival analysis

**Figure 4.**
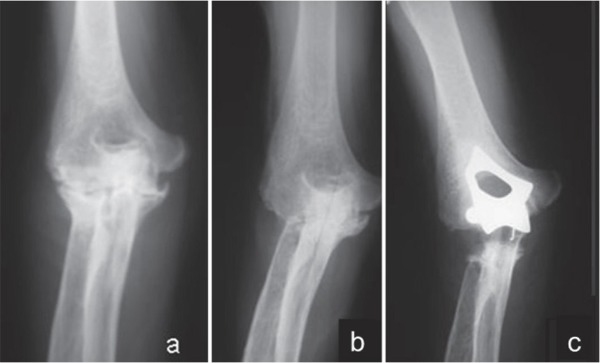
a. Rheumatoid elbow of a 65-year-old woman before synovectomy. b. 13 years after the synovectomy. ADL scores were worse 10 years after the synovectomy. TEA was performed due to the recurrence of arthritis 13 years after the synovectomy. The MEPS was 50 points just before the TEA. c. After TEA, the patient experienced relief of the pain with no swelling and disability in ADL. The MEPS was 80 points at the final follow-up examination, 10 years after TEA.

**Figure 5.**
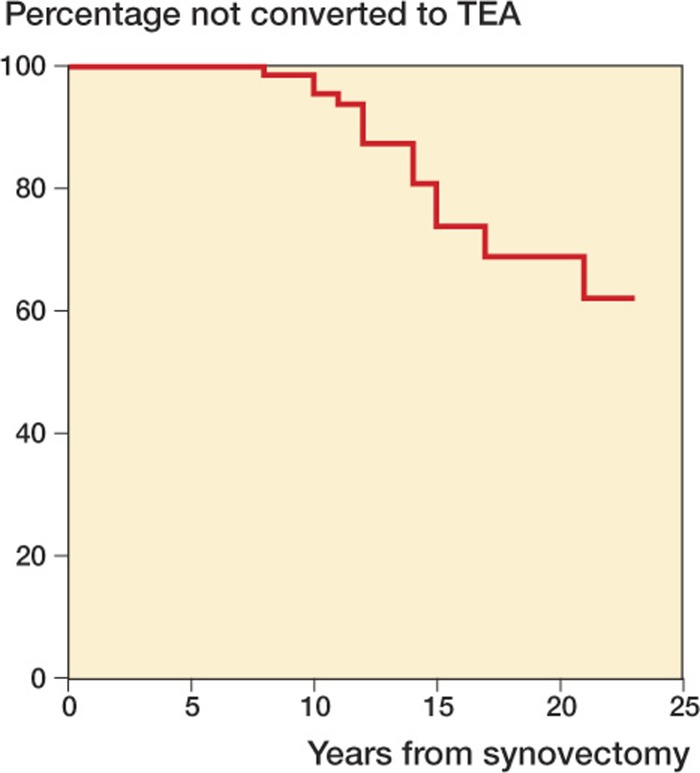
Kaplan-Meier survival curve of synovectomy for rheumatoid elbow with elbows converted to TEA as endpoint. The 10-, 15-, and 20-year survival rates were 96.8%, 74.9%, and 69.9%, respectively.

12 elbows had seen converted to TEA at the final follow-up (Figure 4). Of these cases, 1 elbow had been graded as Larsen grade 3, 7 as grade 4, and 4 as grade 5 preoperatively. The mean MEPS in the TEA conversion cases before synovectomy was 40 (20–60) points, which was not significantly different from that in the cases who did not undergo TEA (43 points). The survival rates for synovectomy were 97% at the 10-year point, 75% at the 15-year point, and 70% at the 20-year point (Figure 5).

### Postoperative complications

There were no cases of postoperative infection. None of the cases needed an additional synovectomy. Ulnar nerve palsy was observed in 4 cases, which all recovered spontaneously and completely. No cases had any loss of motor function. The mean valgus angle of the elbow was 16 (4–28) degrees before surgery and it progressed slightly to 17 (6–33) degrees at the final follow-up examination.

## Discussion

The efficacy of synovectomy for joints at radiographically early stages and TEA for joints at radiographically advanced stages has been confirmed repeatedly (Brumfield and Resnick 1985, Stein et al. 1975, Saito 1986, Ferlic et al. 1987, Figgie et al 1989, Tulp and Winia 1989, Koshino 1991, Gendi et al. 1997, Connor and Morrey 1998, Fuerst et al. 2006, Nakagawa et al. 2007). Our cases underwent open elbow synovectomy, but several reports suggest the efficacy of arthroscopic synovectomy for rheumatoid elbow at radiographically early stages (Lee and Morrey 1997, Horiuchi et al. 2002, Tanaka et al. 2006). However, there have been few reports on long-term outcome of arthroscopic synovectomy (Tanaka et al. 2006), and several authors have reported a relatively high incidence of complications such as local nerve impairment (Lee and Morrey 1997). Thus, we performed open synovectomy to resect the synovium sufficiently and safely.

Medium-term results of elbow synovectomy with an average follow-up period of up to 10 years have been published by several authors. Ferlic et al. (1987) reported excellent results with an average follow-up of 7 years and a maximum follow-up of 20 years. In their report, 44/57 elbows had excellent results, and they had better clinical results in patients who underwent surgery at earlier stages of the disease. Brumfield and Resnick (1985) stated that synovectomy was not contraindicated even for joints at radiographically advanced stages, such as Larsen grade 3 or 4, because an improvement in the range of motion can be expected. We also performed elbow synovectomy at advanced stages and this was not followed by substantial joint instability. In the present study, 12/16 elbows required conversion to TEA after a minimum of 10 years and an average of 15 years of follow-up. We consider this result to be more favorable than those in previous reports that included synovectomies for elbows at radiographically advanced stages (Ferlic et al. 1987, Fuerst et al. 2006). Recurrence of severe pain from progressive joint destruction was the most common cause of the conversion to TEA: 4 of 7 elbows of Larsen grade 5 underwent conversion.

During an elbow synovectomy, the radial head is often resected. Resection of the radial head enhances the operative visual field and the performance of an adequate resection of the joint synovium. Additionally, resection of the radial head improves flexion, especially in cases with anterior subluxation of the radial head. However, resection of the radial head during an elbow synovectomy is controversial. Ferlic et al. (1987) reported that there was no difference in clinical results between cases that had resection of the radial head and those with resection and radial head replacement. Lehtinen et al. (2001) reported that the elbow seemed to turn into valgus during rheumatoid destruction and resection of the radial head. Rymaszewski et al. (1984) suggested that resection of the radial head caused joint instability in the long term, and recommended radial head replacement rather than radial head resection. In addition, Taylor et al. (1976) and Copeland and Taylor (1979) reported that resection of the radial head caused progression of valgus deformities of the elbow in more than half of their cases. They also found that excessive axial pressure to the ulna by resection of the radial head caused pain on the ulnar side of the wrist. We resected the radial head in all our cases with only a slight increase in the valgus angle at the final follow-up examination (mean 2 degrees). At the final examination, only 2 elbows showed marked valgus deformities with severe instability of the elbow joint. Thus, we believe that resection of the radial head is appropriate.

One of the limitations of our study was the evaluation of joint instability, which was assessed clinically, not radiographically. Radiographic examination is a more precise method.

In conclusion, synovectomy for the treatment of rheumatoid elbow gives a good long-term outcome, even for radiologically advanced joints of Larsen grades 3 or 4, but not for those of grade 5.
